# A Review and Future Perspective on Renal Outcome Definitions in Lupus Nephritis

**DOI:** 10.1002/acr2.90099

**Published:** 2026-07-15

**Authors:** Ioannis Parodis, Tina Kochar, Frank Cortazar, Yoshiya Tanaka, Nehad Soloman

**Affiliations:** ^1^ Division of Rheumatology, Department of Medicine Solna Karolinska Institutet, Karolinska University Hospital Stockholm Sweden; ^2^ Center for Molecular Medicine Stockholm Sweden; ^3^ Department of Rheumatology, Faculty of Medicine and Health Örebro University Orebro Sweden; ^4^ Glomerular Disease Program, Department of Internal Medicine University of Texas Medical Branch Galveston; ^5^ St. Peter's Hospital Albany New York; ^6^ New York Nephrology Vasculitis and Glomerular Center Albany; ^7^ Department of Molecular Targeted Therapeutics, First Department of Internal Medicine, School of Medicine University of Occupational and Environmental Health Kitakyushu Japan; ^8^ Yavapai Regional Medical Center Prescott Arizona

## Abstract

Lupus nephritis (LN) remains a leading cause of morbidity and mortality in systemic lupus erythematosus, yet the lack of standardized definitions for renal outcomes hinders effective diagnosis, prognosis, treatment personalization, comparisons across trials, and interpretable trial endpoints. Despite significant advances in biomarker discovery, treatment strategies, and histopathological classification, substantial challenges and controversies persist in defining and assessing renal response to therapy. Following a January 2025 roundtable discussion on the subject of LN outcome definitions, a workgroup was established to address these issues and provide an expert perspective on the future of outcome definitions based on current literature. Here, we examine the current limitations of renal outcome definitions and advocate for change toward improved interpretation and comparison of clinical study data. Key areas, such as limitations of traditional kidney function markers and the need for reliable endpoints reflecting improvements in renal inflammation in trials, are addressed. As an international workgroup of rheumatologists and nephrologists with experience in managing LN, we advocate for a unified approach considering standard per‐protocol repeat biopsies for histologic response evaluation, estimated glomerular filtration rate slope, glucocorticoid minimization/withdrawal, and extended follow‐up for the evaluation of sustained long‐term outcomes. The importance of harmonizing clinical and histologic definitions and the potential of novel biomarkers are highlighted. Herein, we provide a perspective toward uniform definitions of response in LN trials, renal flare/relapse, and refractory disease with improved applicability, especially in the context of advanced immunosuppressives. Thereby, we aim to facilitate meaningful study and advanced treatment of LN, ultimately enhancing patient outcomes and future research.

## Introduction

Lupus nephritis (LN) remains one of the most severe and potentially life‐threatening manifestations of systemic lupus erythematosus (SLE), affecting up to 60% to 70% of patients diagnosed with the disease.^1^, ^2^ LN can manifest as a range of renal pathologies, from mild inflammation to aggressive crescentic disease, and is one of the leading causes of morbidity and mortality in patients with SLE.^3^ It is estimated that 10% to 30% of LN cases result in kidney failure.^4^ Prevalence and severity of LN clearly vary by race and ethnicity, being more prevalent in Hispanic, Black, and Asian populations, with lower rates in European populations.^5^, ^6^, ^7^ Black and Hispanic patients also tend to have more aggressive disease (ie, higher rates of kidney failure).^6^, ^7^ SLE commonly affects women of childbearing age, with active LN and persistent proteinuria being strongly associated with poor maternal–fetal outcomes.^8^ LN tends to develop early during the course of SLE, often within the first five years of diagnosis, with pediatric cases showing even higher prevalence.^9^ Active LN prevalence remains challenging to estimate because it fluctuates over time. Up to 50% of patients with LN relapse even after successful treatment of an initial episode.^10^, ^11^, ^12^ Unfortunately, the diagnosis and management of LN present ongoing challenges, particularly due to the heterogeneous nature of the disease, its variable presentation, and the complexities associated with treatment monitoring.^13^ Patient education is important, as also indicated in the EULAR recommendations for the nonpharmacological management of lupus.^14^

Distinct management challenges due to heterogeneous factors influencing pathogenesis and pathophysiology have contributed to a lack of standardized definitions for treatment response, flares, and refractory disease. Current clinical study definitions are inconsistent and often not applicable to real‐world practice. There is a critical need for a unified terminology to guide both clinical care and research. Standardized definitions would improve consistency across trials, support regulatory alignment, and help clinicians assess treatment effectiveness. These definitions should consider a diverse and interdisciplinary medical perspective. For these reasons, the authors, an international panel of nephrologists and rheumatologists, convened to develop a viewpoint on uniform definitions of treatment response, relapse, and flare to promote clear and applicable targets for disease management and research. We present this viewpoint and future perspective herein.

## Need for updates and consensus

Stratifying LN according to severity and predicting patient outcomes also remain difficult. The risk of progression to kidney failure can vary significantly, depending on the histologic classification of LN and the degree of inflammation and fibrosis present.^15^ This necessitates more reliable markers for prognosis and treatment response.

Although current treatment strategies include glucocorticoids, immunosuppressants, and biologics, there is a pressing need to personalize the use of these therapies based on the patient's specific disease profile. Toward application of such approaches, novel biomarkers, including urinary markers and molecular profiling, may prove useful to better guide therapeutic decisions.^16^, ^17^ In the context of these advances, there is a need for consensus on how to incorporate markers into clinical practice, which is paramount in reaching the goal of improved outcomes. In addition, there is a need for improved recognition of LN as a manifestation of systemic disease. Hence, treatment for LN should also consider ongoing extrarenal disease activity, including joint inflammation, skin disease and cytopenias, among other manifestations.

The optimal timing of kidney biopsy remains an area of uncertainty in LN. Several studies have demonstrated that significant active inflammatory glomerular lesions may be present even in patients with very low levels of proteinuria, or in some cases in the absence of proteinuria altogether.^18^, ^19^, ^20^ These findings challenge proteinuria‐based thresholds for biopsy and reinforce the rationale for early diagnostic evaluation and timely initiation of therapy to prevent irreversible renal damage.

In an effort to standardize the diagnosis and treatment of LN, several important sets of guidelines and definitions have been proposed. However, consensus is lacking because definitions vary among guidelines published by the EULAR,^21^, ^22^ American College of Rheumatology (ACR),^23^ Kidney Disease: Improving Global Outcomes, Spanish Group for the Study of Glomerular Diseases, and others.^5^, ^13^, ^24^ These guidelines have contributed to a more structured approach to diagnosing and treating LN; however, response definitions vary substantially: urinary protein‐to‐creatinine ratio (UPCR) thresholds range from 0.2 to 0.7 g/g across guidelines.^22^, ^25^, ^26^, ^27^, ^28^, ^29^

The International Society of Nephrology/Renal Pathology Society (ISN/RPS) classification system for LN, revised in 2018, clarified definitions regarding histopathological classification of glomerular lesions in LN to improve management of disease based on severity and risk for kidney failure.^5^, ^15^ Limitations in the reproducibility and prognostic value of the ISN/RPS classification system and National Institutes of Health (NIH) activity and chronicity indices have been addressed by proposed consensus recommendations put forth by an expert panel of nephropathologists, clarifying definitions of mesangial hypercellularity, endocapillary hypercellularity, active versus chronic lesions, global versus segmental designation, and inclusion of tubulointerstitial lesions or fibrosis.^5^ However, there is still a need for refinement of the classification of LN to address limitations in current classification systems, for example the inadequate characterization and scoring of tubulointerstitial and vascular lesions.^30^

## Renal outcome definitions

### Limitations of traditional markers

Although clinical measures like hematuria, proteinuria, and serum creatinine levels are commonly used to define response, these markers are not sufficient to capture the full scope of renal function or inflammation in LN. In fact, a 2022 study found that approximately a third of patients classified as complete responders continued to have progressive renal damage as measured using estimated glomerular filtration rate (eGFR) slope despite resolution of proteinuria.^31^ Also, attaining low levels of proteinuria can be made impossible by chronic damage despite a complete immunologic response. This variability complicates the definition of “response” to therapy and necessitates more individualized approaches to treatment. For instance, patients with class IV LN (diffuse proliferative LN) may require more aggressive immunosuppressive therapies compared to those with class V (membranous) disease, but both conditions can still present with similar levels of proteinuria.^32^ Therefore, proteinuria needs to be interpreted in the context of the underlying lesion. Proteinuria can also lack correlation with response because of its reduction through nonimmune mechanisms of some therapeutic agents, such as calcineurin inhibitors.^5^, ^33^ An example of such nonimmune mechanisms is podocyte stabillization.^34^ Despite limitations, proteinuria remains to be recognized as a meaningful early marker and is often predictive of long‐term renal outcomes as in the ALMS trial.^35^

Serum creatinine can be influenced by muscle mass, hydration status, and medications, limiting its specificity as a kidney function marker.^36^ It is also rather insensitive, requiring a large loss of kidney function before a change in creatinine can be appreciated. The use of urinary sediment comes with issues pertaining to menstruation, infection, or trauma, which can give false positives. There is also a somewhat arbitrary nature of what qualifies as hematuria (eg, five red blood cells [RBCs] per high‐power field [hpf]), and there are issues with reproducibility in a clinical trial setting.

### Harmonizing clinical and histologic definitions

Kidney biopsies are also the gold standard for assessing LN activity and chronicity.^15^ Their inconsistent correlation with changes in clinical markers highlights the necessity of an integrative assessment strategy.^30^, ^37^, ^38^ Malvar et al^39^ showed that a third of patients who had a complete clinical response following induction nevertheless had high histologic activity. Likewise, nearly two‐thirds of those with histologic remission after rebiopsy remained clinically active. Incorporating histologic findings alongside biomarker trends can provide a more accurate measure of disease progression and treatment response. A 2020 study showed that high activity indices on per‐protocol repeat biopsies at 24 months were associated with both higher probability of and shorter time to renal relapse, which was independent of proteinuria findings.^33^ De Rosa et al^40^ showed that patients with LN who were in clinical remission but had persistent histologic activity on repeat kidney biopsy were at substantially increased risk of renal flare after withdrawal of long‐term maintenance therapy, highlighting that subclinical inflammation can persist despite prolonged treatment and predict relapse.

A recent Lupus Nephritis Trials Network systematic review aimed to establish standardized biopsy‐based response definitions based on evidence from existing studies that employed repeat biopsies, focusing on correlations between histologic findings and long‐term renal outcomes.^41^ Standardized criteria for histologic remission and response were proposed, incorporating activity and chronicity indices, presence of residual inflammation, and tubulointerstitial damage: response, NIH activity index (AI) score decrease ≥50% by 12 ± 3 months with score ≤3; remission, NIH AI score = 0; and poor prognosis, NIH AI score ≥4. These definitions enhance risk stratification and treatment guidance beyond clinical markers. Per‐protocol repeat biopsies should be incorporated into trial designs to harmonize clinical and histologic endpoints.

### Duration of follow‐up

LN is known to take years to resolve, and maintenance therapy is prolonged due to the risk of relapse, requiring maintenance immunosuppressive therapy that is well tolerated for more than three years.^21^, ^22^, ^38^ The 2025 ACR LN guidelines conditionally recommend a duration of three to five years for immunosuppressive therapy beyond hydroxychloroquine for patients achieving a complete response.^23^ Additionally, kidney damage continues to accumulate early on in treatment despite a therapeutic response.^42^ Therefore, short‐term studies may fail to capture meaningful renal outcomes. Observational studies suggest that a minimum follow‐up period of two years is required to adequately assess sustained renal response.^26^ However, some experts advocate for follow‐up durations of at least five years to distinguish between transient improvements and true sustained remission.^43^ Long‐term follow‐up, particularly in regard to low‐grade immunosuppression, is important for minimizing the risk of late relapses.

### GFR slope as a clinical trial endpoint

Preservation of kidney function is the main goal of therapy. GFR slope is increasingly recognized as a robust measure of kidney function, surpassing absolute spot values in predictive utility, and as a key metric in clinical trials due to its ability to reflect both progression in chronic kidney disease and response to treatment.^25^ Unlike static eGFR measurements, which may not capture subtle changes in kidney function, GFR slope provides a dynamic and continuous assessment, improving the ability to predict long‐term outcomes. Moreover, incorporating albuminuria trends alongside GFR slope analysis may enhance risk prediction models and optimize therapeutic strategies.

The use of eGFR slope as a prognostic tool in IgA nephropathy (IgAN) has garnered significant attention in recent research.^44^ Multiple studies have investigated its utility in predicting disease progression and informing treatment strategies. In a retrospective cohort study, the first‐year eGFR slope was found to be a significant predictor of renal outcomes in patients with IgAN.^45^ Patients were categorized based on their eGFR decline rates: rapid decliners, slow decliners, and nondecliners. The study demonstrated that rapid decliners had a significantly higher risk of renal progression compared to nondecliners, which was independent of baseline proteinuria and histologic findings.

Further supporting the prognostic value of eGFR slope, a 2019 meta‐analysis evaluated data from 47 randomized controlled trials encompassing over 60,000 participants. The analysis revealed that treatment effects on the eGFR slope were strongly associated with clinical outcomes, suggesting that eGFR slope could serve as a surrogate endpoint in clinical trials for chronic kidney disease.^46^ Moreover, a 2021 study highlighted the relationship between early changes in proteinuria and subsequent eGFR decline in patients with IgAN. The findings indicated that reductions in proteinuria were predictive of slower eGFR decline, emphasizing the interconnectedness of these two markers in disease progression.^47^ It is important to note potential limitations in using eGFR slope as a surrogate endpoint. There are complexities in interpreting eGFR slope changes, especially in the context of treatments that may cause acute effects on kidney function, which could confound the assessment of long‐term disease progression.^44^

### Glucocorticoid sparing as an endpoint

Long‐term glucocorticoid exposure is associated with significant morbidity, including osteoporosis, diabetes, and cardiovascular disease.^48^ Cumulative prednisone exposure in patients with lupus is independently associated with increased permanent organ damage, even after accounting for underlying disease activity.^49^, ^50^, ^51^, ^52^ In a study by Thamer et al,^49^ higher glucocorticoid doses were linked to worse long‐term outcomes, indicating that prednisone itself contributes to irreversible harm rather than merely reflecting more severe disease. These findings underscore the importance of minimizing glucocorticoid exposure in LN to improve long‐term prognosis and reduce treatment‐related morbidity. The overall organ damage associated with SLE, along with that associated with the disease activity of SLE, becomes more pronounced over time due to the organ damage caused by glucocorticoid use. If successful treatment with immunosuppressants is measured by decreased kidney inflammation, there will be a correlation with a decreased need for glucocorticoids. ACR conditionally recommends tapering prednisone to ≤5 mg daily by six months of treatment.^23^ Therefore, clinical trials should prioritize glucocorticoid‐sparing regimens as a key measure of treatment success. The development of novel biologics, including B cell–targeted therapies and calcineurin inhibitors, has enabled more effective glucocorticoid‐minimizing treatment approaches.^53^, ^54^ Developing alternative immunosuppressive strategies that maintain efficacy while reducing glucocorticoid dependence is crucial for improving patient quality of life.

## Future perspectives on unified renal response definitions

Given the evidence discussed herein, the working group advocates for uniform definitions of response that hierarchically consider histologic score (standardized per‐protocol repeat kidney biopsies), followed by clinical response if histology is unavailable, as exemplified in Table 1. Trial endpoints reflecting treatment goals should include preservation of kidney function using eGFR slope, novel immunologic markers that correlate with histologic response and glucocorticoid minimization (prednisone ≤5 mg in the four weeks preceding the assessment time point) as appropriate. The use of response cutoffs generally results in a loss of informational granularity. In this regard, proteinuria and eGFR trajectories, or their slopes over time, may be more informative and carry greater predictive value. Proteinuria and renal function measures may be more meaningful if persisting for at least 18 months; however, a customized approach based on patient response is justified. Such clinical measures are supported by the adoption of similar measures in major trials such as AURORA (voclosporin), BLISS‐LN (belimumab), and NOBILITY (obinutuzumab), which were informative regarding outcome.^25^, ^28^, ^55^, ^56^ The AURORA 1 trial demonstrated that voclosporin, when added to mycophenolate mofetil and low‐dose glucocorticoids, significantly increased the rates of complete renal response (CRR) rates at 52 weeks (40.8% vs 22.5% with placebo, *P* < 0.001).^57^ Voclosporin can cause a slight downward bump in the eGFR but still preserves the eGFR in the long term, based on AURORA long‐term extension trial.^58^ This initial decline need not be a reason for waiting to introduce therapy with voclosporin (or tacrolimus) as we usually do for angiotensin‐converting enzyme inhibitors because the rapid effect of calcineurin inhibition on proteinuria is important to limit mechanistic renal toxicity during the early phases of treatment when proteinuria usually is high. The REGENCY trial showed improved CRR with obinutuzumab over placebo (46.4% vs 33.1%, *P* = 0.02).^55^ The NOBILITY trial also showed that obinutuzumab increased CRR at 104 weeks compared to placebo (38% vs 22%, *P* = 0.06), although not to statistical significance, and reduced renal flare risk by 57% over two years.^25^ In the BLISS‐LN trial of belimumab, 30% achieved CRR compared to 20% with placebo (odds ratio 1.7, 95% confidence interval 1.1–2.7; *P* = 0.02).^56^ It is important to recognize that many patients with LN, particularly those from lower socioeconomic strata with suboptimal insurance coverage, face barriers to timely specialty care, including prolonged delays in securing appointments with nephrologists and other subspecialists, which can complicate the incorporation of timely biopsies.

**Table 1:** Existing clinical response criteria and potential unified hierarchical criteria for histologic and clinical definitions of renal response*

Definition	Existing criteria	Hierarchical criteria
Complete response	KDIGO: Reduction in UPCR <0.5 g/g (50 mg/mmol) measured as the PCR from a 24‐hour urine collection Stabilization or improvement in kidney function (±10%–15% of baseline) Within 6–12 months of starting therapy but could take more than 12 months; for children <18 years old, proteinuria < 0.5 g/1.73 m^2^/day or <300 mg/m^2^/day^a^	NIH AI score = 0 on repeat biopsy or UPCR < 0.5 g/g^a^ or 24‐hour protein excretion < 0.5 g/day and stable (within ±15% of baseline) or improved eGFR
	ACR: UPCR < 0.2 g/g Normal Scr, 25% improvement in eGFR if abnormal with LN flare Inactive urine sediment EULAR/ERA–European Dialysis and Transplant Association: Proteinuria < 0.5g/24 hours and a near‐normal eGFR	
Partial response	KDIGO: Reduction in proteinuria by at least 50% and to <3 g/g (300 mg/mmol) measured as the PCR from a 24‐hour urine collection Stabilization or improvement in kidney function (±10%–15% of baseline) Within 6–12 months of starting therapy	NIH AI score decreases by ≥50% by 12 ± 3 months with a score ≤3 on repeat biopsy or ≥50% reduction in UPCR from baseline, but not achieving <0.5 g/g^a^ and stable (±15% of baseline) or improved eGFR
	ACR: UPCR of 0.2–2.0 g/g eGFR at baseline or improved by 25% if abnormal with LN flare Inactive urine sediment EULAR/ERA–European Dialysis and Transplant Association ≥50% reduction in proteinuria and a near‐normal eGFR	
No renal response	KDIGO: Failure to achieve a partial or complete response within 6–12 months of starting therapy	Failure to meet complete response or partial response criteria within 12 months
	ACR: No change in or worsening proteinuria >10% increase in eGFR from baseline EULAR/ERA–European Dialysis and Transplant Association: Failure to achieve a partial or complete response within 6–12 months of starting therapy	

* ACR, American College of Rheumatology; AI, activity index; eGFR, estimated glomerular filtration rate; ERA, European Renal Association; KDIGO, Kidney Disease: Improving Global Outcomes; LN, lupus nephritis; NIH, National Institutes of Health; PCR, protein‐to‐creatinine ratio; Scr, serum creatinine; UPCR, urinary PCR.

^a^ Whenever a biopsy is not possible, clinical targets can be applied.

Novel urinary immune markers, such as monocyte chemotactic protein 1 (MCP‐1), CD163, and neutrophil gelatinase–associated lipocalin (NGAL), correlate with histologic response and may be more informative, particularly when immunosuppressives are evaluated. We envision a trend toward use of novel urinary immune markers, such as MCP‐1, CD163, and NGAL, which correlate with histologic response and may be more informative, particularly when immunosuppressives are evaluated and as evidence accumulates. It is desirable to identify reliable biomarkers that reflect intrarenal activity and may ultimately reduce the need for repeat kidney biopsies. However, such markers are not yet available, largely because repeat biopsy material has been scarce, limiting access to the gold‐standard information needed to anchor peripheral biomarkers to intrarenal pathology. For this reason, repeat kidney biopsies currently remain necessary, also for research purposes, because they provide a crucial foundation for identifying markers that may eventually form a clinically useful liquid biopsy biomarker panel.

## Future perspectives on definitions of renal disease status

### Renal flare/relapse

There has been variability in used definitions of renal flare, which typically include a combination of increased proteinuria, hematuria, and/or declining renal function.^59^ Renal flare is an increase in disease activity that requires augmentation of therapy. Historically, the terms renal flare and relapse have been used interchangeably, but some experts argue that they should have distinct definitions. For instance, renal flare may refer to the first episode of kidney involvement in a patient with SLE, whereas relapse implies a recurrence of renal activity following a period of remission or control, although both terms are indicative of an increase in renal SLE activity necessitating an adjustment in therapy.^60^

Another key discussion point is whether relapse implies a prior successful response to therapy. Renal relapse is often referred to as a flare in a patient with prior disease activity who had previously achieved disease quiescence.^43^ If a patient had an incomplete response to initial treatment and subsequently worsened, the term “relapse” may not be entirely appropriate. Although distinguishing between flare and relapse has no practical utility in clinical decision‐making, it may be relevant for research and regulatory purposes.

Definitions of flares have traditionally incorporated different elements, such as proteinuria, serum creatinine, and urinary sediment. For instance, an increase in glomerular hematuria (>15 RBCs per hpf with acanthocytes) or recurrence of RBC/white blood cell (WBC) casts has been used to signify mild flare,^43^, ^61^ while an increase in serum creatinine of 0.2 to 1.0 mg/dL if baseline was <2 mg/dL or an increase in serum creatinine of 0.4 to 1.5 mg/dL if baseline was ≥2 mg/dL is seen in definitions of moderate flare.^62^ In the BLISS trials of belimumab, renal flare was defined as the occurrence of one or more specific criteria on two or more consecutive visits during the study period. First, it included a reproducible increase in 24‐hour urine protein levels beyond certain thresholds relative to baseline: >1 g if the baseline was less than 0.2 g, >2 g if the baseline was between 0.2 and 1 g, or more than twice the baseline value if it was greater than 1 g. Second, a reproducible rise in serum creatinine by at least 20% or ≥0.3 mg/dL, accompanied by proteinuria (greater than 1 g/24 hours), hematuria (≥4 RBCs per hpf), or the presence of RBC casts, was considered a renal flare. Third, emergent and reproducible hematuria (≥11–20 RBCs per hpf) or a two‐grade increase in hematuria compared to baseline, associated with at least 25% dysmorphic RBCs of glomerular origin (excluding menses), also met the criteria. This condition had to be accompanied by either an increase in 24‐hour proteinuria by ≥0.8 g or the appearance of new RBC casts.^54^

Renal flare in LN can also be defined using the British Isles Lupus Assessment Group 2004 index, grading kidney‐specific disease activity based on change in serum creatinine, UPCR, hematuria, RBC/WBC casts, presence of nephrotic syndrome, and histologic confirmation. Grade A indicates very active disease, such as biopsy‐confirmed nephritis, new or worsening nephrotic syndrome, or a rise in serum creatinine of more than 30% attributable to lupus, whereas grade B reflects moderately active disease, such as proteinuria with a PCR between 50 and 100 mg/mmol or a creatinine rise of less than 30%.^63^ A renal flare is typically identified when a patient's renal domain score shifts to a higher activity grade, signifying increased disease activity.

Pappa et al^64^ described EULAR/European Renal Association–European Dialysis and Transplant Association–aligned definitions of renal flares in the context of immunosuppressive treatment, which were classified as proteinuric, defined by an increase in proteinuria to >1 g/24 hr if a prior complete response had been achieved or >2 g/24 hr if only a partial response had been achieved, or nephritic, defined by increased hematuria (>10 RBCs per hpf) with a concurrent rise in serum creatinine (>30% or ≥10% decline in eGFR). Although GFR‐based elements in definitions of flare exist, they require refinement because serum creatinine changes may be an unreliable marker of kidney function fluctuation.^65^ Serologic markers such as complement levels and anti–double‐stranded DNA are useful but are alone insufficient.^66^ Histologic inflammation can persist for months to years, underscoring the limitations of clinical metrics in defining flares.^67^ Furthermore, proteinuria variability due to diet, salt intake, or concurrent medications complicates flare definitions.^68^

### Refractory renal disease

The definition of refractory LN also remains debated. Some define it as active LN with an inadequate response to initial therapy.^69^ Others propose that refractory status should be reserved for patients who fail multiple therapies.^60^ The variability in definitions has led to an ongoing Delphi survey within the Lupus Nephritis Trials Network, which aims to establish consensus criteria. A key concern is distinguishing true treatment resistance from poor adherence, suboptimal dosing, or inadequate monitoring. Although current therapies and their incorporation into guidelines have reduced the use of glucocorticoids, they carry a high pill burden, which can create challenges for all populations but especially those with lower health literacy or non‐English speakers. This can further complicate good renal outcomes. One study found less than 25% adherence to immunosuppressive therapy for SLE, which was defined as refill of medication greater than 80% of the time.^70^ Drug level monitoring often reveals inadequate medication intake rather than true treatment failure.^71^ This highlights the need for careful evaluation before categorizing a patient as refractory because this classification may lead to the escalation of treatment to aggressive or experimental therapies. Overall, patient‐centered strategies should be optimized.

The importance of standardized definitions extends beyond semantics because they have practical implications for treatment decisions and clinical trial design. For example, novel therapies, including chimeric antigen receptor T cell therapy, often target “refractory” patients, yet concerns exist that some enrolled patients may be undertreated rather than truly refractory. This highlights the need for precise inclusion criteria in clinical trials to ensure investigational therapies are tested in appropriate patient populations.

Additionally, the nature of LN treatment presents challenges in defining refractory disease. Current therapies do not cure the disease but aim to control it, and many patients experience flares upon tapering immunosuppressive medications.^72^ This raises the question of whether these patients are truly refractory or experiencing the expected disease course. Biomarkers and other objective measures may be required to refine treatment decisions beyond traditional clinical parameters such as proteinuria and serum creatinine.^73^

Standardized definitions for renal flare, relapse, and refractory disease are needed for the reasons discussed above. Although distinctions exist, their clinical utility remains debated due to the heterogeneity of LN presentations and responses to treatment. Future efforts should aim to establish expert consensus criteria.^30^ Such efforts are crucial for improving patient care, optimizing clinical trial design, and ensuring that emerging therapies are appropriately targeted to the patients who need them most. A viewpoint on potential unified definitions of flare, relapse, and refractory renal disease is given in Table 2.

**Table 2:** Flare, relapse, and refractory renal disease*

Domain	Definition	Key elements
Renal flare	Reproducible increase in renal disease activity requiring therapeutic intensification on at least two consecutive measurements	Proteinuria: UPCR ≥ 1.0 g/g if previously <0.5 g/g or at least two‐fold increase from documented nadir Kidney function: ≥25% rise in serum creatinine from baseline or nadir, confirmed on repeat testing
Renal relapse	Renal flare occurring after a documented partial or complete response	Same criteria as flare, with the additional requirement of prior response documentation
Refractory renal disease	Persistent active lupus nephritis despite adequate trials of at least two standard therapy regimens with confirmed adherence and therapeutic dosing	Failure to achieve at least partial response after appropriate duration Exclusion of nonadherence, subtherapeutic drug exposure, or misclassification Consideration of biopsy or biomarker evidence when feasible

* UPCR, urinary protein‐to‐creatinine ratio.

## Conclusions

LN remains a significant cause of morbidity and mortality in SLE, yet the lack of standardized renal outcome definitions continues to hinder clinical management, trial comparability, and treatment personalization. Current challenges include the previously discussed limitations of traditional biomarkers such as proteinuria and serum creatinine, as well as inconsistencies in defining response, flare, and refractory disease across guidelines. This workgroup advocates for a unified set of definitions and an eye toward incorporating GFR slope, glucocorticoid minimization, and extended follow‐up periods to better assess long‐term outcomes. Additionally, harmonizing clinical and histologic definitions, including the potential role of repeat biopsies and novel biomarkers, is essential for accurately capturing disease progression and therapeutic response. By advocating for improved consensus on these key parameters, we aim to enhance the interpretability of clinical trials, improve patient outcomes, and facilitate the development of more effective therapies.

Moving forward, the integration of advanced biomarkers, dynamic measures like GFR slope, and histologic validation will be critical in refining LN management. The variability in current definitions of complete and partial response, relapse, and refractory disease underscores the need for standardized criteria that align with long‐term renal preservation. Furthermore, the increasing use of advanced immunosuppressive therapies necessitates outcome measures that reflect true disease modification rather than transient improvements. Collaborative efforts among clinicians, researchers, and regulatory bodies will be essential to implement these changes, ensuring that future trials yield meaningful and comparable data. Ultimately, a consensus‐driven framework for LN outcomes will not only optimize clinical decision‐making but also accelerate the development of targeted therapies, improving the prognosis for patients with this challenging complication of SLE. New, more potent therapies, such as cell therapies, will change expectations, and definitions will need to be reconsidered soon, taking into account these new modalities.

Although standardized, biomarker‐ and biopsy‐driven approaches to LN management are appealing goals, important real‐world barriers must be acknowledged. Access, even to current standard‐of‐care therapies, remains inconsistent. For example, calcineurin inhibitors such as voclosporin are unavailable in some countries (eg, Canada), and publicly insured patients in underresourced US settings face challenges related to medication access and timely subspecialty follow‐up. In this context, the routine use of high‐cost urinary biomarker panels and per‐protocol repeat kidney biopsies may place substantial financial and logistical strain on health systems. As a result, these advanced strategies may currently be feasible only in well‐resourced, highly academic centers.

This article is based on literature searches last conducted from January to August 2025 and opinion of the workgroup. It is intended to support decision‐making but is not intended to establish a standard of care or dictate a singular approach to management. Clinical practices will naturally vary as health care providers consider individual patient needs, available resources, and institutional or practice‐specific constraints. Professionals using this information should determine how best to incorporate it into their own clinical practice.

## AUTHOR CONTRIBUTIONS

All authors contributed to at least one of the following manuscript preparation roles: conceptualization AND/OR methodology, software, investigation, formal analysis, data curation, visualization, and validation AND drafting or reviewing/editing the final draft. As corresponding author, Dr Parodis confirms that all authors have provided the final approval of the version to be published and takes responsibility for the affirmations regarding article submission (eg, not under consideration by another journal), the integrity of the data presented, and the statements regarding compliance with institutional review board/Declaration of Helsinki requirements.

## ROLE OF THE STUDY SPONSOR

Aurinia Pharmaceuticals had no role in the study design or in the collection, analysis, or interpretation of the data or the decision to submit the manuscript for publication. Publication of this article was not contingent upon approval by Aurinia Pharmaceuticals.

## Supporting information


**Disclosure form**.
